# Self-report symptom-based endometriosis prediction using machine learning

**DOI:** 10.1038/s41598-023-32761-8

**Published:** 2023-04-04

**Authors:** Anat Goldstein, Shani Cohen

**Affiliations:** 1 Department of Industrial Engineering and Management, Ariel University, 65 Ramat HaGolan St., Ariel, Israel; 2 Department of Computer Science, Ariel University, 65 Ramat HaGolan St., Ariel, Israel

**Keywords:** Risk factors, Diagnosis, Diseases, Information technology

## Abstract

Endometriosis is a chronic gynecological condition that affects 5–10% of reproductive age women. Nonetheless, the average time-to-diagnosis is usually between 6 and 10 years from the onset of symptoms. To shorten time-to-diagnosis, many studies have developed non-invasive screening tools. However, most of these studies have focused on data obtained from women who had/were planned for laparoscopy surgery, that is, women who were near the end of the diagnostic process. In contrast, our study aimed to develop a self-diagnostic tool that predicts the likelihood of endometriosis based only on experienced symptoms, which can be used in early stages of symptom onset. We applied machine learning to train endometriosis prediction models on data obtained via questionnaires from two groups of women: women who were diagnosed with endometriosis and women who were not diagnosed. The best performing model had AUC of 0.94, sensitivity of 0.93, and specificity of 0.95. The model is intended to be incorporated into a website as a self-diagnostic tool and is expected to shorten time-to-diagnosis by referring women with a high likelihood of having endometriosis to further examination. We also report the importance and effectiveness of different symptoms in predicting endometriosis.

## Introduction

Endometriosis is a chronic gynecological condition that affects 5–10% of women of reproductive age^1,2^. Women with endometriosis have endometrial-type tissue outside of the uterus^1,3^. In exceptional cases, endometriosis lesions may reach organs distant from the pelvis such as the membranes of the lungs, heart, limbs, and brain. As a result, and in response to the substances that this tissue produces, the immune system is activated, and a chronic inflammatory process is triggered, leading to the formation of adhesions, scars, and cysts between the pelvic and abdominal organs. Endometriosis tissue can also penetrate various organs in the body, including the digestive and urinary systems, and attach to nerves^4,5^.

Endometriosis is associated with a wide variety of symptoms such as pain, abnormal bleeding, gastrointestinal symptoms, urinary system problems, and even emotional effects^2,4,6^. This variety, together with a lack of awareness, may explain the relatively long duration until the condition is typically diagnosed: currently, the average time-to-diagnosis of women suffering from the disease is about 6–10 years from symptom onset^7^.

Usually, an endometriosis diagnosis includes a pelvic exam, ultrasound imaging of reproductive organs, an MRI, and laparoscopy. These tests are expensive and invasive and require the involvement of a physician. The literature recognizes the need for non-invasive screening tools to simplify the diagnostic process and shorten time-to–diagnosis^8,9^, and various studies have investigated the feasibility of several non-invasive tools. One example of such non-invasive indicators are biomarkers obtained from blood-tests^10–13^. For example, Nisenblat et al.^12^ reviewed works that combined non-invasive blood tests and transvaginal ultrasound to improve the diagnostic accuracy of pelvic endometriosis. However, they found that the accuracy obtained in those works was insufficient to replace laparoscopy. Another non-invasive tool whose effectiveness for endometriosis prediction has been studied is genomic data^14–18^. Studies have identified several biomarker genes that are indicative of endometriosis^14^ and developed ML-based models for endometriosis prediction^14,15^. The use of patient-reported symptoms is another non-invasive approach that has been investigated in previous studies. However, most of these studies have incorporated not only symptoms, but also imaging and clinical parameters, which are often available only in later diagnosis stages, are costly, and require the involvement of physician^5,19–21^. In fact, in a review study, Surrey et al.^19^ found only one study that used a questionnaire based exclusively on patients’ self-reported symptoms^22^. This study applied multiple logistic regression to subfertile women undergoing laparoscopy and analyzed the associations between seven self-reported symptoms and endometriosis. However, only one symptom, period pain, was found to be significantly different between women with endometriosis and women with a normal pelvis.

In recent years, machine learning (ML) has been used as a promising approach for patient classification, with excellent results in various medical fields^23–27^. ML has also been used for endometriosis prediction and diagnosis^3,14,15,24,28,29^. Indeed, ML is promising because it facilitates the discovery of complex, non-linear relationships between a set of variables (such as patient characteristics or symptoms) and a target variable (such as the patient’s likelihood of having endometriosis). A recent review by Sivajohan et al.^3^ found 36 studies that applied ML in endometriosis prediction, diagnosis, and research. Only three of these studies^6,24,30^ used self-report questionnaires to develop ML-based models for endometriosis prediction. However, in addition to symptoms experienced, these models also included clinical data, which were available since these studies focused on women who underwent or were scheduled for laparoscopy, that is, women who were in advanced diagnosis stages and could provide such data.

Our research, in contrast, aims to serve women who are only beginning their medical investigative journey and who have not yet received any test results or formal diagnosis. For this population, we develop an easy-to-use self-diagnostic tool based exclusively on self-reported symptoms, rather than on information that is available to women who went through medical investigation.

Thus, the main goal of the presented research is to develop an ML-based model that predicts the likelihood of having endometriosis based on patient-completed questionnaires, in which they report their experienced symptoms. Such a model is intended to serve as a preliminary tool for self-test, which women can take to provide them with indication or likelihood for having endometriosis. A second goal is to identify a sufficient subset of symptoms that are most relevant for endometriosis prediction.

Our investigation generated a set of 24 symptoms that were found to be most effective for endometriosis prediction. This model obtained sensitivity of 0.93 and specificity of 0.95 on holdout data. The developed model is intended to be incorporated into a website that offers women a questionnaire they can complete about the symptoms they experience and that returns their likelihood of having endometriosis. The model and the website are expected to shorten the currently long time-to-diagnosis. We also offer insights on the importance of the different symptoms and their effectiveness in predicting endometriosis.

## Materials and methods

### Data collection

To collect the data for our endometriosis prediction model, we distributed a survey (see Supplementary Information) via Facebook to women over the age of 18 who were and were not diagnosed with endometriosis. To reach women with endometriosis, we distributed the survey in Facebook groups dedicated to women who suffer from the disease. Members of these groups included women from Europe, United States, Australia, and Israel, however, no demographic information related to respondents’ age or ethnicity was recorded to maintain respondents’ anonymity.

The survey included 56 endometriosis symptoms that were compiled based on an extensive review of relevant literature^2,5,7,19–22,30–34^. Respondents indicated (true/false) whether they experienced each symptom in the past month. Informed consent was obtained from all responders and that the study was approved by the ethics committee of Ariel University and performed in accordance with all relevant guidelines and regulations.

### Descriptive statistics analysis

We started with model-free analysis to better understand the frequency of symptom occurrence in the two groups of women (diagnosed/undiagnosed). We used chi-square tests to investigate the differences between the frequencies of each symptom in the two groups. A large difference between the two groups in the occurrence rate of a symptom indicates the symptom’s predictive power of endometriosis.

### Machine learning

We applied several ML algorithms to train multiple endometriosis prediction models. Specifically, we applied decision trees, Random Forest, Gradient Boosting Classifier (GBC), and Adaptive Boosting (AdaBoost). Besides generating predictions, these models also provide an importance analysis feature, which can be used to identify and remove non-contributing features from future surveys.

Model performance was evaluated using common ML metrics: accuracy, sensitivity (recall), specificity, precision, F1-score, and area under the ROC curve (AUC). To ensure significance of the results, we used a ten-fold cross-validation procedure.

### Machine learning algorithms

We applied several ML algorithms to train four types of classification models:

- Decision Tree classifier—This is a simple, tree-structured classifier, where internal nodes represent the features of a dataset, branches represent the decision rules, and each leaf node represents the outcome (class). The tree structure (organization of nodes) is determined based on the importance of the nodes using an attribute selection measure, such as information gain or Gini index^35,36^. The model’s simplicity is both its weakness and its strength: On the one hand, this model is limited in its capacity to capture complex relationships between variables, yet on the other hand, its classification process is simple to interpret.
- Random Forest classifier—This model generates a “forest” of decision trees, such that each tree is trained on a random subset of the features. The Random Forest model uses the entire collection of decision trees to classify a given sample, and eventually determines the classification output based on the trees’ majority vote, that is, the class that is the output of by most trees^37,38^.
- Gradient Boosting Tree classifier—This model is an ensemble of multiple decision trees (weak learners) that are added together to create a strong predictive model. In the training process of this model, trees are added to the model in an effort to minimize the error of the model, as in a gradient descent procedure. Gradient Boosting models are known to be effective at classifying complex datasets^39^.
- Adaptive Boosting (AdaBoost) classifier is a boosting technique used as an ensemble method. It is called adaptive because weights are reassigned to each sample such that higher weights are assigned to incorrectly classified samples^40^.

### Symptom importance analysis

Based on the descriptive statistics and feature (symptom) importance obtained by the trained models, we analyzed each symptom’s contribution to the model’s ability to correctly classify women. We also analyzed the correlation between the symptoms. A high correlation may indicate that a symptom is redundant. Because symptom values are binary (indicate whether a respondent does or does not experience the symptom), we use the Jaccard index^41^, which is commonly applied for measuring similarity between two binary datasets (in our case, representing symptom values). 

To further analyze the importance of the different symptoms in the various types of models, we extracted from each model its feature importance ranking (we used the built-in ‘*feature_importances_*’ attribute of scikit-learn classifier classes). We then trained and tested each model using its first *n* important symptoms, where n = 1, 2, …, 56 (using ten-fold cross-validation), and compared each symptom’s contribution to the model’s performance, in order to identify the optimal set of symptoms.

## Results

### Descriptive statistics

In total, 886 responders completed the survey. Of these, 474 had a diagnosis of endometriosis and 412 had no diagnosis, that is, did not undergo a diagnostic procedure. We note that it is possible that some proportion of the undiagnosed women suffer from endometriosis but have not yet been diagnosed. Such respondents may introduce bias into our model and cause false negatives. Nevertheless, as the percentage of endometriosis is estimated between 5 and 10%, we expect such bias to be relatively small.

Table 1 presents descriptive statistics of the symptoms, including their frequency, that is, the percentage of women who suffer from each symptom (mean value) in each group (1—with endometriosis, 0—without), the absolute difference between the mean values, and the p-values (chi-square-test) indicating the significance of mean differences. Symptoms are listed in descending order of absolute mean differences. Table 1 also includes symptom importance according to an AdaBoost model.

**Table 1:** Descriptive statistics that indicate the importance of each symptom.

Symptom	Not diagnosed	Diagnosed	Absolute mean diff	P-value (χ^2^-test)	Importance (AdaBoost)
Menstrual pain (dysmenorrhea)	0.05 (0.05)	0.76 (0.18)	0.71	0.0	0.04
Cramping	0.23 (0.17)	0.83 (0.14)	0.60	2.03E−289	0.024
Painful cramps during period	0.07 (0.07)	0.67 (0.22)	0.60	3.87E−221	0.032
Fatigue/chronic fatigue	0.11 (0.1)	0.7 (0.21)	0.59	5.58E−211	0.03
Heavy/Extreme menstrual bleeding	0.2 (0.16)	0.77 (0.17)	0.59	3.11E−215	0.054
Pelvic pain	0.19 (0.15)	0.76 (0.18)	0.57	0.0	0.034
Abdominal pain/pressure	0.11 (0.1)	0.67 (0.22)	0.56	1.28E−123	0.018
Painful/Burning pain during intercourse (dyspareunia)	0.12 (0.11)	0.67 (0.22)	0.55	0.0	0.022
Back pain	0.28 (0.2)	0.77 (0.18)	0.49	1.76E−112	0.016
Bloating	0.14 (0.12)	0.62 (0.24)	0.47	0.0	0.022
Lower back pain	0.15 (0.12)	0.62 (0.24)	0.47	7.74E−146	0.016
Sharp/stabbing pain	0.08 (0.08)	0.54 (0.25)	0.45	0.0	0.004
Painful bowel movements	0.05 (0.05)	0.51 (0.25)	0.45	5.97E−154	0.038
Pain/chronic pain	0.16 (0.13)	0.61 (0.24)	0.45	1.25E−55	0.022
Decreased energy/exhaustion	0.14 (0.12)	0.58 (0.24)	0.44	3.07E−184	0.002
Stomach cramping	0.09 (0.08)	0.53 (0.25)	0.44	0.0	0
Menstrual clots	0.04 (0.04)	0.47 (0.25)	0.42	4.84E−29	0
Ovarian cysts	0.01 (0.01)	0.43 (0.25)	0.42	0.0	0.022
Irregular/missed periods	0.09 (0.08)	0.49 (0.25)	0.40	3.02E−155	0.044
Painful ovulation	0.12 (0.11)	0.53 (0.25)	0.40	1.96E−237	0.028
Nausea	0.17 (0.14)	0.56 (0.25)	0.39	2.74E−14	0.006
Extreme/severe pain	0.11 (0.1)	0.5 (0.25)	0.39	1.58E−165	0.022
Pain after intercourse	0.07 (0.06)	0.45 (0.25)	0.39	1.23E−42	0
Hormonal problems	0.07 (0.06)	0.42 (0.24)	0.36	2.64E−115	0.026
Anxiety	0.18 (0.15)	0.53 (0.25)	0.35	8.22E−188	0.016
Cysts (unspecified)	0.02 (0.02)	0.37 (0.23)	0.35	1.90E−68	0.02
Constipation/chronic constipation	0.04 (0.04)	0.39 (0.24)	0.35	1.97E−58	0.016
IBS-like symptoms	0.02 (0.02)	0.36 (0.23)	0.34	1.30E−208	0.034
Vaginal pain/pressure	0.09 (0.08)	0.42 (0.24)	0.33	1.22E−188	0.02
Mood swings	0.2 (0.16)	0.53 (0.25)	0.32	3.06E−70	0.018
Abdominal cramps during Intercourse	0.06 (0.05)	0.38 (0.23)	0.32	0.0	0.02
Digestive/GI problems	0.06 (0.05)	0.36 (0.23)	0.30	2.71E−122	0
Long menstruation	0.05 (0.05)	0.35 (0.23)	0.30	1.18E−30	0.012
Depression	0.2 (0.16)	0.5 (0.25)	0.30	5.22E−59	0.002
Acne/pimples	0.09 (0.09)	0.39 (0.24)	0.29	5.47E−244	0
Infertility	0.06 (0.05)	0.33 (0.22)	0.27	7.23E−179	0.02
Diarrhea	0.17 (0.14)	0.44 (0.25)	0.27	0.0	0
Anaemia/iron deficiency	0.07 (0.06)	0.33 (0.22)	0.27	9.81E−123	0.002
Feeling sick	0.2 (0.16)	0.46 (0.25)	0.26	1.59E−51	0.02
Painful urination	0.06 (0.06)	0.32 (0.22)	0.26	2.74E−141	0
Leg pain	0.2 (0.16)	0.45 (0.25)	0.25	9.12E−282	0.004
Irritable Bowel Syndrome (IBS)	0.06 (0.05)	0.3 (0.21)	0.25	5.28E−43	0.016
Hip pain	0.15 (0.12)	0.39 (0.24)	0.24	7.79E−91	0.002
Insomnia/sleeplessness	0.17 (0.14)	0.41 (0.24)	0.24	0.0	0
Headaches	0.25 (0.19)	0.49 (0.25)	0.23	2.45E−42	0.02
Dizziness	0.16 (0.13)	0.39 (0.24)	0.23	2.17E−12	0.008
Bowel pain	0.14 (0.12)	0.35 (0.23)	0.22	9.70E−21	0.038
Fertility issues	0.05 (0.05)	0.23 (0.18)	0.18	2.22E−08	0.022
Migraines	0.3 (0.21)	0.46 (0.25)	0.16	2.34E−218	0.002
Vomiting/constant vomiting	0.1 (0.09)	0.26 (0.19)	0.16	1.61E−191	0.018
Loss of appetite	0.2 (0.16)	0.34 (0.22)	0.14	6.34E−45	0.03
Constant bleeding	0.03 (0.03)	0.17 (0.14)	0.13	3.91E−168	0.028
Syncope (fainting, passing out)	0.01 (0.01)	0.14 (0.12)	0.13	3.67E−191	0
Fever	0.23 (0.18)	0.12 (0.11)	0.11	8.67E−44	0.024
Abnormal uterine bleeding	0.04 (0.04)	0.13 (0.11)	0.09	3.71E−104	0.042
Malaise/Sickness	0.08 (0.07)	0.16 (0.13)	0.09	1.74E−14	0.024

For each symptom we present the percentage (mean and variance in parenthesis) of undiagnosed and diagnosed women who experience the symptom, the absolute mean (frequency) difference between undiagnosed and diagnosed women, and whether the difference is significant (chi-square test, p-value < 0.01). The rightmost column presents the importance of each symptom according to the AdaBoost model, which is detailed below.

### Endometriosis classification models

Four types of classification models were trained: Decision Tree, Random Forest, Gradient Boosting and Adaptive Boosting (AdaBoost). Table 2 summarizes the performance of these models. To ensure significance, we used a ten-fold cross-validation procedure, and we report the mean and standard deviation (in parentheses) of the following performance metrics: recall (sensitivity), specificity, precision, F1-score, accuracy, and AUC.

**Table 2:** Classification models performance metrics.

	1 Decision Tree	2 Random Forest	3 Gradient Boosting	4 AdaBoost
Recall (sensitivity)	0.890 (0.035)	0.924 (0.029)	0.924 (0.02)	0.939 (0.029)
Specificity	0.859 (0.039)	0.937 (0.031)	0.932 (0.051)	0.934 (0.052)
Precision	0.880 (0.029)	0.945 (0.026)	0.942 (0.042)	0.944 (0.042)
F1-score	0.885 (0.019)	0.934 (0.02)	0.932 (0.021)	0.941 (0.029)
Accuracy	0.876 (0.02)	0.930 (0.022)	0.928 (0.024)	0.937 (0.032)
AUC	0.875 (0.02)	0.930 (0.022)	0.928 (0.025)	0.937 (0.033)

This table shows the predictive performance across four classification models (1) Decision tree, (2) Random Forest, (3) Gradient Boosting, (4) AdaBoost. For each metric we present the mean value and standard deviation based on ten-fold cross-validation.

We find while all models demonstrate high performance, the AdaBoost achieves the best results with AUC and accuracy of 94%.

### Symptom importance

Table 1 presents symptom occurrence frequency by group. A large difference in a symptom’s frequency between the two groups indicates that the symptom may be effective for an endometriosis diagnosis classification. The rows in Table 1 are sorted by the absolute difference between group means (frequencies) in descending order of symptoms’ importance for classification. Although, as seen in Table 1, all differences are statistically significant (all p-values are smaller than 0.01), the symptoms (features) at the bottom of the table may be non-contributing and may even cause overfitting of the models.

**Figure 1:**
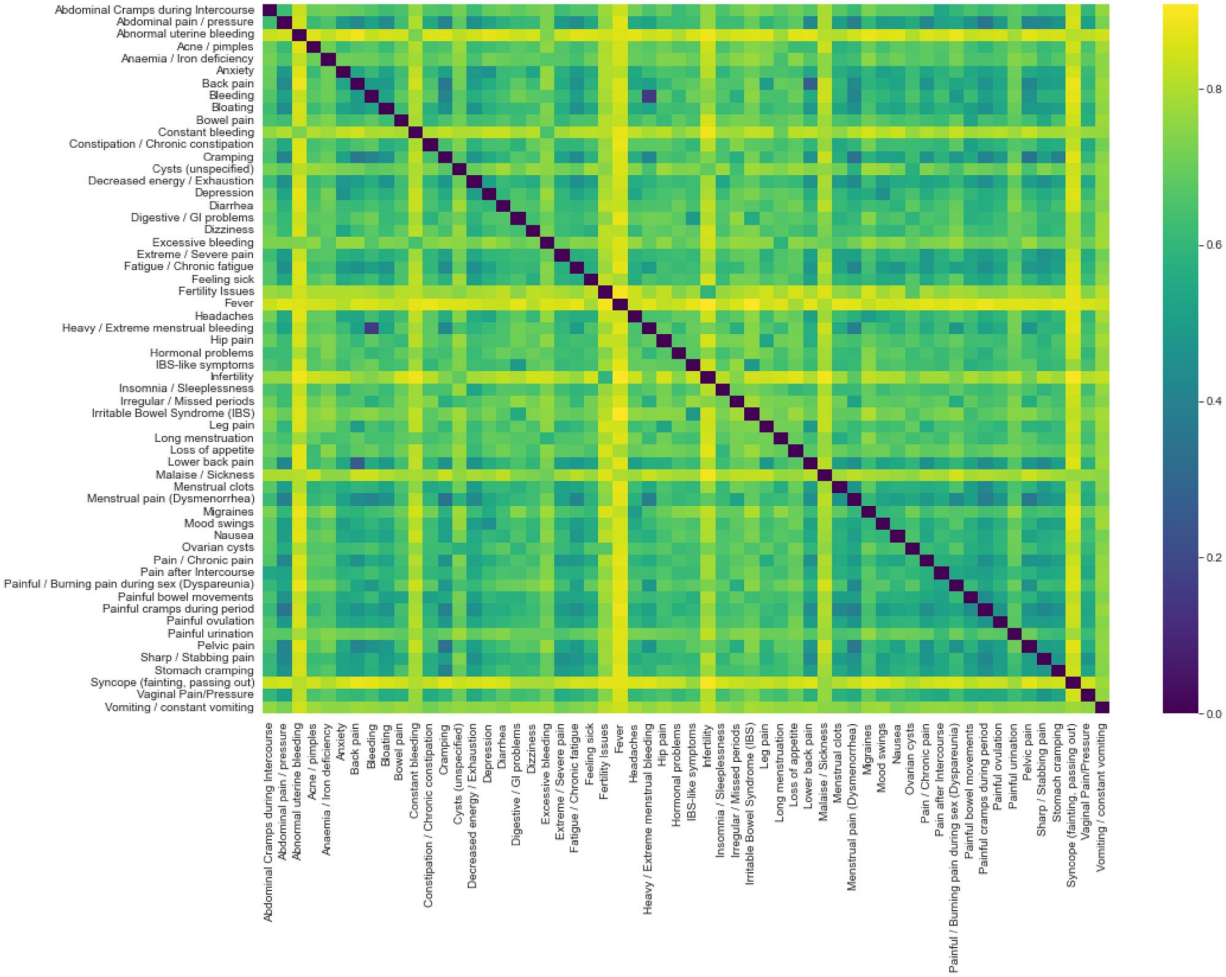
A heatmap that shows Jaccard Indices between each pair of symptom value vectors. A darker color indicates a lower Jaccard Index distance, or a strong similarity between symptoms. We use the Jaccard Index to identify potentially redundant symptoms.

High correlations between symptoms may indicate redundancy. To identify symptoms that are highly correlated with other symptoms, we calculated the correlation between each pair of symptom values using the Jaccard Index. Figure 1 shows a heatmap of the Jaccard Index distance values, derived as 1-Jaccard index, which reflect the correlation levels between symptom pairs. In this figure, darker cells signify smaller distances, indicating a higher degree of similarity between the symptoms. Notably, all calculated Jaccard distance values exceeded 0.25, and following this analysis, no columns were eliminated due to redundancy.

**Figure 2:**
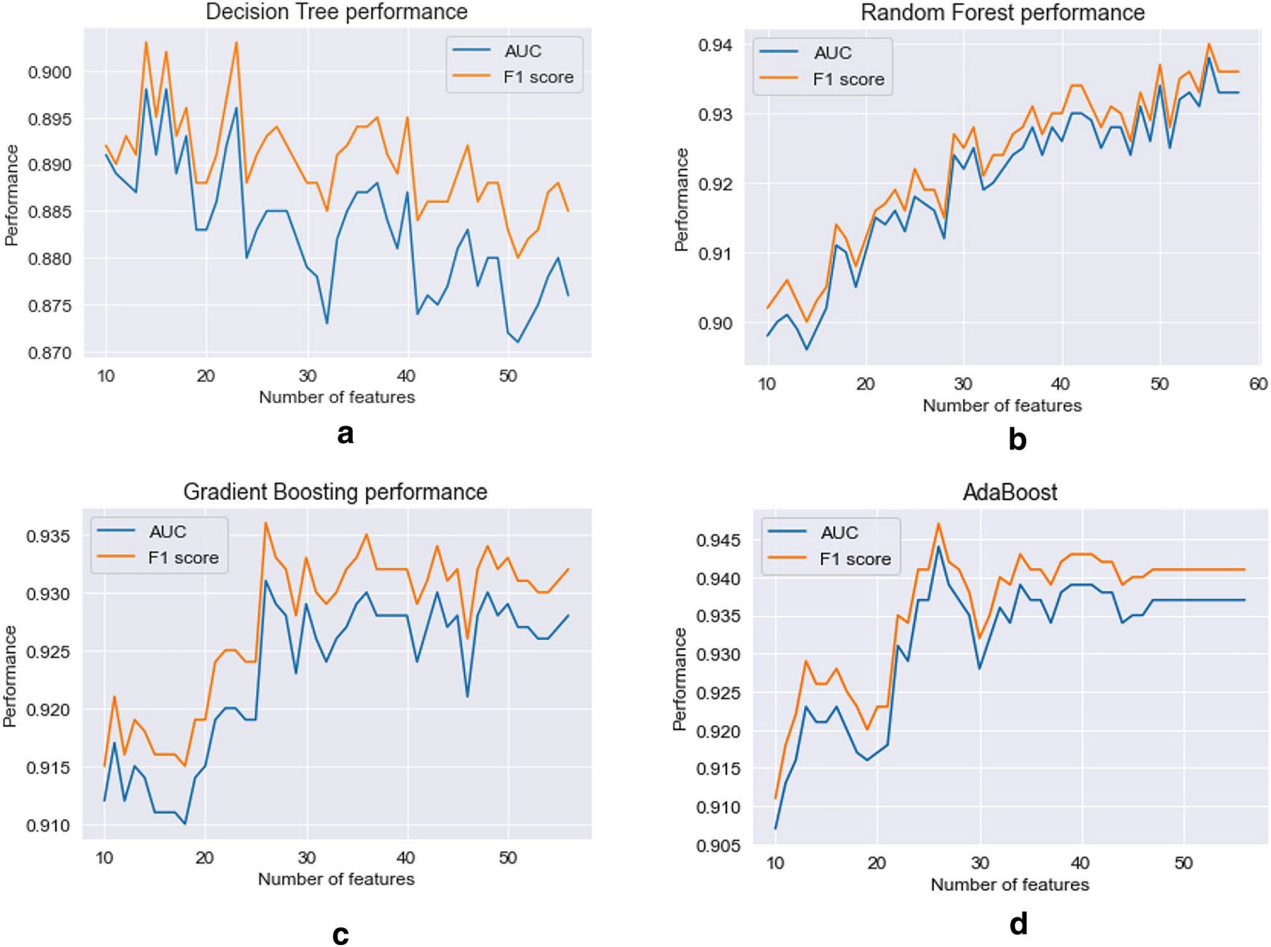
The performance of each model across symptom subsets. Models: (**a**)—Decision Tree, (**b**)—Random Forest, (**c**)—Gradient Boosting Trees, (**d**)—AdaBoost. Features are ordered according to each model’s feature importance.

As discussed above, for each model type we also analyzed the effect of adding each symptom in the order of its importance based on the *feature importance* ranking derived from initial classification models (the models that were trained on the entire set of features, as shown in Table 2). Figure 2 demonstrates the improvement in the performance using AUC and F1-score (ten-fold cross-validation mean values) of the Decision Tree (a), Random Forest (b), Gradient Boosting Trees (c) and AdaBoost (d) models when adding features one by one.

These results provide insights on the performance of each model type and how performance changes when additional symptoms are added to the model. For example, we see that the Decision Trees model generates the best results (AUC of 0.898) when the model includes 14 symptoms, and adding additional symptoms hampers the model’s performance. In contrast, the performance of the Random Forest model improves as symptoms are added to the model, and provides the best performance with 55 symptoms (AUC of 0.938).

For each model type we selected the number of features (n) that yields the best AUC. We then trained each model using only that selected number of features, that is, the n most important features. Table 3 presents the performance metrics of each model (mean values and standard deviations of tenfold cross-validation).

**Table 3:** Performance metrics when including the first *n* important features of each model.

	1 Decision Tree n = 14	2 Random Forest n = 55	3 Gradient Boosting n = 26	4 AdaBoost n = 24
Recall (sensitivity)	0.893 (0.05)	0.926 (0.037)	0.93 (0.024)	0.932 (0.026)
Specificity	0.903 (0.045)	0.949 (0.018)	0.932 (0.046)	0.946 (0.038)
Precision	0.915 (0.036)	0.955 (0.015)	0.942 (0.036)	0.954 (0.032)
F1-score	0.903 (0.03)	0.94 (0.019)	0.936 (0.019)	0.943 (0.023)
Accuracy	0.897 (0.031)	0.937 (0.019)	0.931 (0.022)	0.939 (0.024)
AUC	0.898 (0.031)	0.938 (0.018)	0.931 (0.023)	0.939 (0.025)

The value of n is indicated in the header of each column. For each metric, we present the mean value and standard deviation based on ten-fold cross-validation.

The AdaBoost model remains the best performing model, with AUC of 93.9%. It is based on only 24 symptoms. Other symptom subsets selected on the basis of criteria other than feature importance, may yield better performance. However, because it is impossible to check all possible subsets, the method used here, based on feature importance, should be effective for identifying relevant subsets of features and for creating optimal models.

To further verify that no additional symptoms should be removed, we iteratively removed each feature, and then retrained and tested all the models. In all cases, performance metrics became worse.

The features included in the best performing model (the 24-feature AdaBoost model) are, in descending order of importance: heavy/extreme menstrual bleeding, irregular/missed periods, abnormal uterine bleeding, menstrual pain (dysmenorrhea), painful bowel movements, bowel pain, pelvic pain, IBS-like symptoms, painful cramps during period, fatigue/chronic fatigue, loss of appetite, constant bleeding, painful ovulation, hormonal problems, malaise, fever, cramping, bloating, painful/burning pain during intercourse (dyspareunia), extreme/severe pain, pain/chronic pain, ovarian cysts, fertility issues, and feeling sick.

### Sample size adequacy

**Figure 3:**
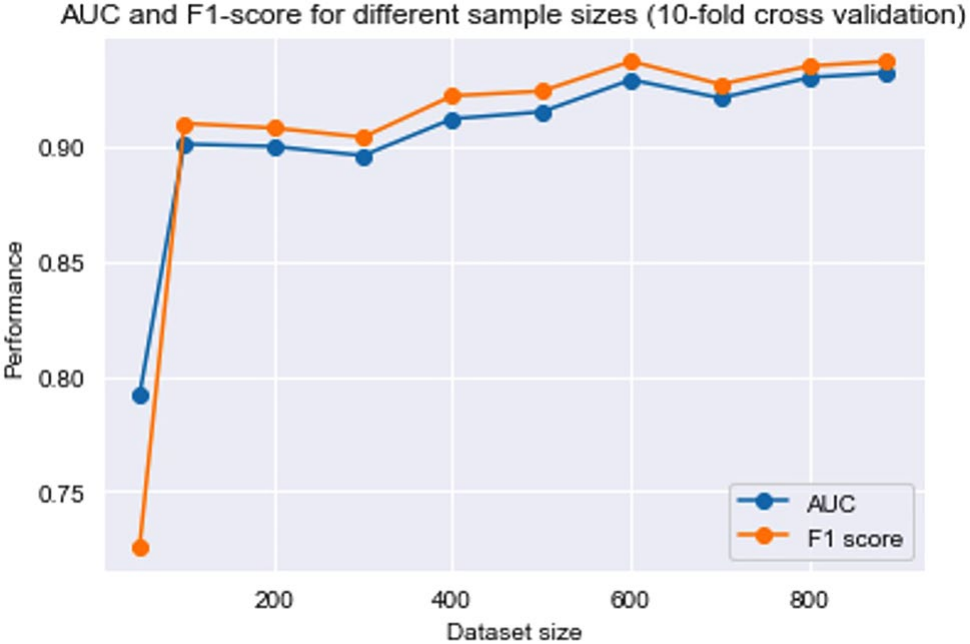
The performance of 24-symptom AdaBoost model when trained on different dataset sizes.

As a robustness check, to confirm that we used an adequate number of samples, we trained the 24-symptom AdaBoost model on different dataset sizes and measured model performance. Figure 3 shows the model’s AUC and F1-score (ten-fold cross-validation means) when trained on different dataset sizes. It shows that adding the dataset samples beyond 600 samples has little effect on the model’s performance and indicates that our sample size is sufficient.

## Discussion and conclusion

In this study, we developed several classification models for endometriosis prediction, based exclusively on self-reported symptoms. We compared four types of classification models, namely, Decision Tree, Random Forest, Gradient Boosting Trees and AdaBoost, and showed that the AdaBoost model obtained the best results, with AUC, accuracy, and F1-score of 0.94; sensitivity of 0.93; and specificity of 0.95. We also applied multiple approaches to analyze the importance of each symptom and found that the best performing AdaBoost model is based on a subset of 24 of the original 56 symptoms.

While numerous studies developed questionnaire-based models and indices to predict or indicate endometriosis, these models include clinical parameters that were correlated with macroscopic/microscopic presence or absence of endometriosis^5,7,21,22^. Other studies investigated the relationship between different symptoms and the likelihood of endometriosis, however most were unable to successfully predict whether a patient has endometriosis^22,31,34,42^. For example, Forman et al.^22^ found that severe period pain (dysmenorrhea) was the single symptom found to be predictive of endometriosis, yet were unable to sufficiently distinguish women with endometriosis from women with a normal pelvis using the questionnaire used in their study. Calhaz-Jorge et al.^34^ focused on subfertile women and found subfertility, dysmenorrhea, chronic pelvic pain, oral contraception use (ever), and obesity (inverse relationship) to be predictive of endometriosis.

Only few studies have employed ML to develop endometriosis prediction models based on self-reported symptoms. As discussed above, ML models can capture complex and non-linear relationships between a set of independent variables and a target variable and are thus expected to be effective for linking between sets of symptoms and endometriosis diagnosis. Most of the models developed in these studies were trained on data that were collected from women who had or were planned to have laparoscopy^6,7,20,24,30^ and included information that is not available in the early phases of the diagnosis process. For example, Nnoaham et al.^30^ included indications of past surgeries, ultrasound evidence, etc. Their model has sensitivity of 83% and specificity of 76%. Bendifallah et al.^24^ also used patient history and treatment data and developed a model with sensitivity of 93% and specificity of 92% (no information on significance is provided). Chapron et al.^6^ also used previous surgery for endometriosis as a predictor. Their model has sensitivity of 75% and specificity of 69%. Yeung^20^ in contrast, used only standard pain symptoms and quality-of-life questions. They studied women with chronic pelvic pain before surgery and developed a logistic regression model that had sensitivity of 80.5% and specificity of 57.7%.

Nevertheless, classification models that were trained on women in advanced diagnosis stages (e.g.,^6,7,20,24,30^), are expected not to work well when applied to the general population of women at reproductive age. First, these models are expected to learn to give less weight to symptoms experienced by all women who started medical investigation– whether they were eventually diagnosed with endometriosis or not. For example, an ML model that was trained on women with chronic pelvic pain before surgery, will give less weight to the *pelvic pain* symptom, whereas for women in the general population *pelvic pain* is considered a common symptom, which strongly differentiates women with and without endometriosis. Second, because these models also rely on data that were obtained during the diagnosis process (e.g., results of a laparoscopy) and are unavailable to women in the early stage of the diagnostic process, these models may falsely classify women as not having endometriosis because they are missing this information. Thus, a model that intends to serve women who have not yet begun a medical investigation and will be applied to the general population of women, should be trained exclusively on experienced symptoms and only on data that are available to women who are at that point in their medical journey.

Two recent studies developed endometriosis classification models based on symptom-only questionnaires^2,7^. Chapron et al.^7^ applied multiple logistic regressions on pain symptoms and patient data obtained through pre-surgery interviews to predict endometriosis at different stages of the condition, and showed that patient questionnaires can be used to identify women at high risk of endometriosis (sensitivity of 91% for a highly sensitive model and sensitivity of 73% and specificity of 75% in a model that maximizes both sensitivity and specificity). Fauconnier et al.^2^ used a 21-symptom questionnaire on women with endometriosis confirmed by histology, asymptomatic women, and women without endometriosis diagnosis who suffer from pain/infertility. They applied binary logistic regression analysis to predict endometriosis and obtained AUC of 92%.

Similarly to these studies, in our study we also developed models for predicting the likelihood of endometriosis based only on symptoms experienced (or not experienced) by women. Our study differs from these studies in two main respects: First, it uses tree ensemble models, which are able to capture complex and non-linear relationships between the variables. Second, we used Facebook to collect data rather than patient interviews. While this is a convenient way to collect data and allowed us to collect responses from almost 1000 women within a few months, it gives us less information on the respondents.

The developed models, and in particular the 24-feature AdaBoost model, can be self-administered by women who suffer from symptoms and are at the beginning their diagnostic investigation to discover the likelihood that their symptoms are caused by endometriosis. It should, however, be noted that as our models are trained on women who were clinically diagnosed with endometriosis and on women who were not diagnosed (rather than who were clinically found not to have endometriosis), our models may be biased by women who have endometriosis yet were not diagnosed. Nevertheless, since this may affect only a small percentage of the non-diagnosed group, the effect on the models’ classification performance is expected to be relatively small and the best performing model is expected to identify most of those women who have endometriosis. Moreover, had we tested the models on women with a positive or negative clinical diagnosis of endometriosis, the models’ performance would have been even better, as false positive samples would have become true positives.

It should also be noted that our data did not include information on respondents’ demographics (e.g., ethnicity, geographic location, and age) and thus our models did not account for these variables. Future research should validate these models on different populations. Future research should also investigate the effectiveness of the models (i.e., their predictive power) for women at different stages of diagnosis and account for additional variables that are available to women who have not started a medical investigation, such as use of contraception and hormones.

To summarize, the contribution of our study is threefold. First, we developed a questionnaire for self-reporting of endometriosis symptoms based on 56 symptoms that are commonly found in the literature. Second, we analyzed the importance of these symptoms for endometriosis prediction. We also analyzed the frequency of each symptom in the group of women with endometriosis, compared to the frequency in the general population. We further identified a subset of symptoms that provided the highest endometriosis prediction accuracy. Third, we developed a model that is able to predict endometriosis in the general population of women with high accuracy (94%), based on a subset of 24 self-reported symptoms. The developed model is expected to shorten time-to-diagnosis, which is currently 6 to 10 years from symptom onset. Furthermore, the developed model is intended to be incorporated into a website that women can use to self-test themselves and discover their likelihood of suffering from endometriosis. This website is intended to refer women to conduct further examinations for endometriosis at an early stage in the diagnostic investigation.

### Supplementary Information

Supplementary Information.

## Data Availability

The data and code used in the current study are available from the corresponding author upon reasonable request.
