# Author Correction: Self-report symptom-based endometriosis prediction using machine learning

**DOI:** 10.1038/s41598-024-61280-3

**Published:** 2024-05-07

**Authors:** Anat Goldstein, Shani Cohen

**Affiliations:** 1 Department of Industrial Engineering and Management, Ariel University, 65 Ramat HaGolan St., Ariel, Israel; 2 Department of Computer Science, Ariel University, 65 Ramat HaGolan St., Ariel, Israel

Correction to: *Scientific Reports* 10.1038/s41598-023-32761-8, published online 04 April 2023

The original version of this Article contained an error in the interpretation of the heatmap presented as Figure 1.

As a result, in the Results section under the subheading, ‘Symptom importance’,

“Figure 1 shows a heatmap of the Jaccard Index values, indicating the correlation between each pair of symptom values. In this figure, the yellow rows/columns indicate that the symptom is highly correlated with many other symptoms. We identified six symptoms that are highly correlated (Jaccard Index > 0.8) with more than 30% of the symptoms: fever, abnormal uterine bleeding, syncope (fainting, passing out), infertility, constant bleeding, and malaise/sickness. Five of these symptoms appear at the bottom of Table 1. To investigate whether removing these potentially redundant features improves the models’ classification performance, we trained the models again without these six symptoms. Table 3 present the performance results of the different models. After removing the highly correlated symptoms, the performance of the Decision Tree model improved, whereas the performance of the remaining models diminished slightly.”

now reads:

“Figure 1 shows a heatmap of the Jaccard Index distance values, derived as 1-Jaccard index, which reflect the correlation levels between symptom pairs. In this figure, darker cells signify smaller distances, indicating a higher degree of similarity between the symptoms. Notably, all calculated Jaccard distance values exceeded 0.25, and following this analysis, no columns were eliminated due to redundancy.”

Additionally, in section “Symptom importance analysis”, the sentence “We analyzed the performance of the models after removing symptoms that are highly correlated with other symptoms (Jaccard Index close to 1).” was removed.

Thirdly, in the legend of Figure 1:

“A heatmap that shows Jaccard Indices between each pair of symptom value vectors. A lighter color indicates a higher Jaccard Index, or a strong similarity between values. We use the Jaccard Index to identify potentially redundant symptoms.”

now reads:

“A heatmap that shows Jaccard Indices between each pair of symptom value vectors. A darker color indicates a lower Jaccard Index distance, or a strong similarity between symptoms. We use the Jaccard Index to identify potentially redundant symptoms.”

Lastly, Table 3 was removed.

The original Article has been corrected.

