# Supplementary material for: Self-report symptom-based endometriosis prediction using machine learning
Source: Sci Rep. 2023 Apr 4;13:5499. doi: 10.1038/s41598-023-32761-8 (PMC10073113; doi:10.1038/s41598-023-32761-8)
Supplement: Supplementary file 1 — Supplementary Information. [file 41598_2023_32761_MOESM1_ESM.pdf]

Supplementary Information

Self-Report Symptom-Based Endometriosis Prediction using Machine Learning

Anat Goldstein*[1] and Shani Cohen[2]

[1] Department of Industrial Engineering and Management, Ariel University, 65 Ramat HaGolan St., Ariel, Israel, anatgo@ariel.ac.il.
[2] Department of Computer Science, Ariel University, 65 Ramat HaGolan St., Ariel, Israel

Endometriosis symptom questionnaire

Were you diagnosed with endometriosis?

□ Yes □ No

Please check all the symptoms below that you experienced at least once in the past few months.

□ Heavy / Extreme menstrual bleeding
□ Menstrual pain (Dysmenorrhea)
□ Painful / Burning pain during intercourse (Dyspareunia)
□ Pelvic pain
□ Irregular / Missed periods
□ Cramping
□ Abdominal pain / Pressure
□ Back pain
□ Painful bowel movements
□ Nausea
□ Menstrual clots
□ Infertility
□ Painful cramps during period
□ Pain / Chronic pain
□ Diarrhea
□ Long menstruation
□ Constipation / Chronic constipation
□ Vomiting / constant vomiting
□ Fatigue / Chronic fatigue
□ Painful ovulation
□ Stomach cramping
□ Migraines
□ Extreme / Severe pain
□ Leg pain
□ Irritable Bowel Syndrome (IBS)
□ Syncope (fainting, passing out)
□ Mood swings
□ Depression
□ Lower back pain
□ Fertility issues
□ Ovarian cysts
□ Painful urination
□ Headaches

☐ Constant bleeding
☐ Pain after intercourse
☐ Digestive / GI problems
☐ IBS-like symptoms
☐ Anaemia / Iron deficiency
☐ Hip pain
☐ Vaginal Pain/Pressure
☐ Sharp / Stabbing pain
☐ Bowel pain
☐ Anxiety
☐ Cysts (unspecified)
☐ Dizziness
☐ Malaise
☐ Abnormal uterine bleeding
☐ Fever
☐ Hormonal problems
☐ Bloating
☐ Feeling sick
☐ Decreased energy / Exhaustion
☐ Abdominal Cramps during Intercourse
☐ Insomnia / Sleeplessness
☐ Acne / pimples
☐ Loss of appetite
